# Supplementary figures and images for: Effect of HIV-1 subtype-specific Tat protein polymorphisms on Tat-TAR interaction
Source: PLoS One. 2026 Apr 20;21(4):e0346629. doi: 10.1371/journal.pone.0346629 (PMC13095106; doi:10.1371/journal.pone.0346629)

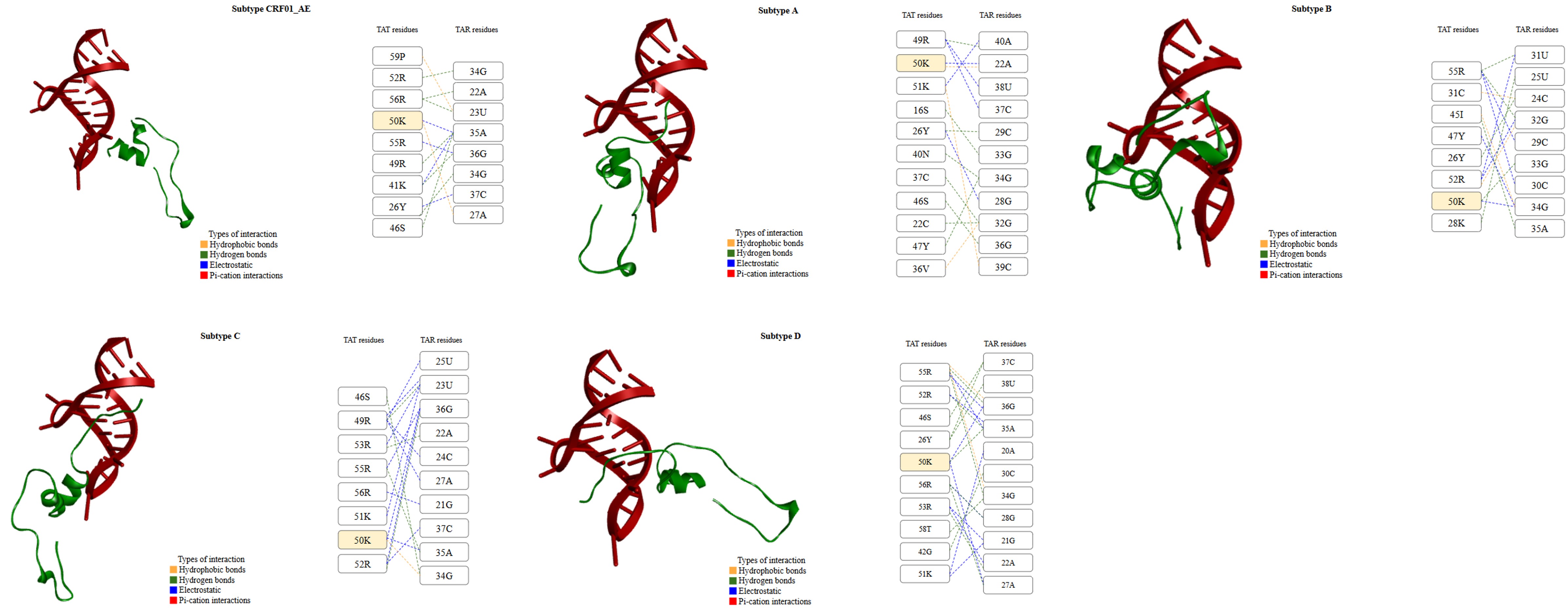

Supplement: S1 Fig — (JPG) [file pone.0346629.s002.jpg]

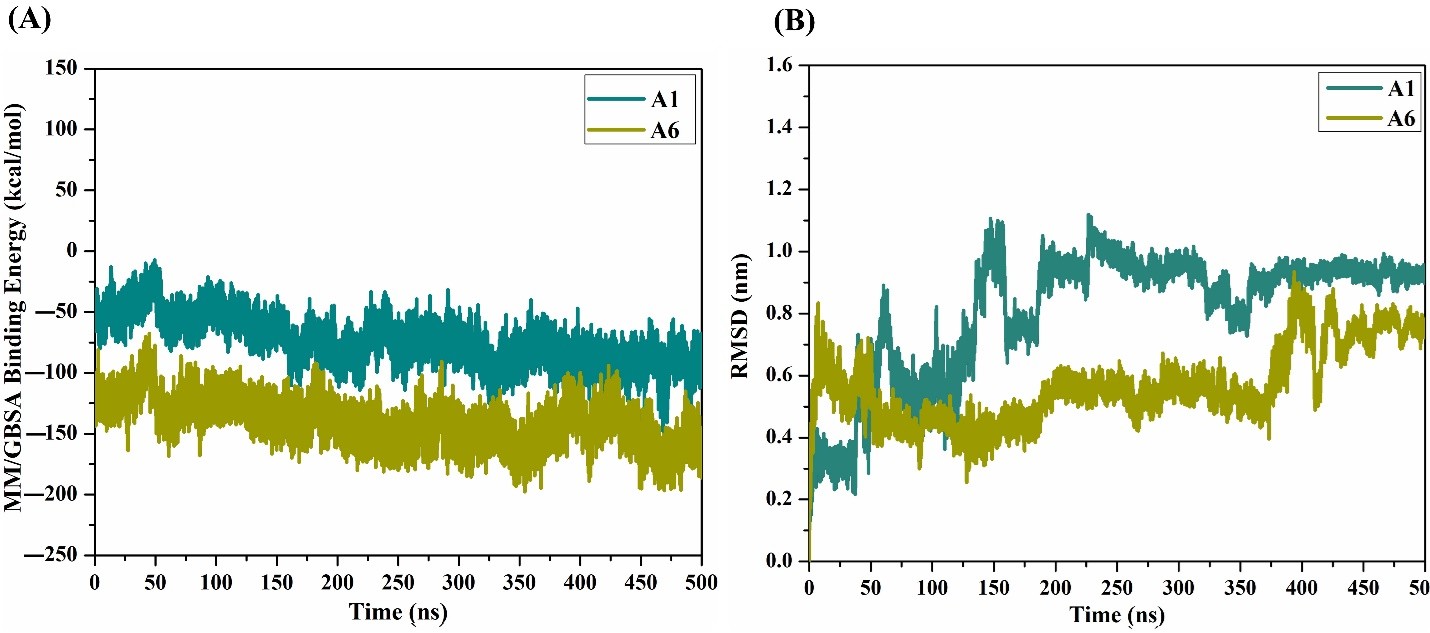

Supplement: S2 Fig — A) binding energy per frame and B) RMSD of the backbone atoms with respect to the initial structure of the Tat protein from different subtypes. Key: A1 (cyan) and A6 (yellow) in complex with TAR RNA over 500 ns MDS. (JPG) [file pone.0346629.s003.jpg]
