# Supplementary material for: Effect of HIV-1 subtype-specific Tat protein polymorphisms on Tat-TAR interaction
Source: PLoS One. 2026 Apr 20;21(4):e0346629. doi: 10.1371/journal.pone.0346629 (PMC13095106; doi:10.1371/journal.pone.0346629)
Supplement: Attachment — Submitted filename: Response_R4.docx [file pone.0346629.s005.docx]

**Response to the Reviewers' Comments:**

The authors would like to thank the Reviewers for their time and effort in reviewing our manuscript. The authors sincerely appreciate all the valuable comments and suggestions, which helped us to improve the quality of the manuscript. We have tried our best to address all comments and suggestions. The reply to each comment is given below, while the changes have been highlighted in yellow in the manuscript.

**Reviewer 1**

**Comment** #**1**: Have you validated subtype-specific Tat protein polymorphisms on functional cell line based assays? please refer and cite to this paper : PMID: 24465566, PMID: 28484443, PMID: 28468838 and PMID: 31652847.

**Reply**: We thank the reviewer for pointing out the issue about the functional validation of Tat polymorphisms. In our paper, we have incorporated findings from PMID: 28484443, PMID: 24465566, PMID: 28468838, and PMID: 31652847. Please see lines can be checked in lines 376-399.

**Comment** **#2**: Have you tested subtype-specific other viral genes like VIf protein polymorphisms ? please refer and cite to this paper : PMID: 26494109. does the subtype-specific Tat protein polymorphisms have any effort on host genes like CCR5? please refer and cite to this paper :PMID: 31110236

**Reply**: We thank the reviewer for suggesting these papers! As suggested, we have incorporated the findings from PMID: 31110236 and PMID: 26494109. Please see lines 92-96 and 376-380.

**Reviewer 2:**

**Comment #1:** A substantial body of literature exists regarding the role of the Tat-TAR axis in HIV. For instance, it demonstrates that Tat is both necessary and sufficient for HIV activity, particularly in the context of establishing and reversing latency through TAR mutations. It seems unlikely that the authors intended to overlook this prior research; discussing these earlier studies would provide important context for their results.

**Reply**: We thank the reviewer for the comment, and we agree that discussing the role of the Tat-TAR axis in latency provides important context for our findings. Thus, we have addressed this point in the revised manuscript, lines 128-136. We have reviewed key studies demonstrating Tat's role in establishing and reversing viral latency. We believe that the impact of genetic variations in Tat across subtypes (aim of our paper) beyond B and C on the Tat-TAR interaction remains underexplored.

**Comment #2**: The authors should clarify whether the TAR sequences differ among the HIV subtypes they examined.

**Reply**: We used a single and highly conserved TAR sequence across all subtypes analysis to ensure that observed differences in binding were attributable specifically to Tat protein polymorphisms. Please see lines 178-180.

**Comment #3**: It is important to consider the disease stage of the subtypes when modeling the predictions and structures.

**Reply**: We thank the reviewer for raising this point. We completely agree that disease stage is a critical factor when interpreting functional consequences of Tat polymorphisms. In the revised manuscript, we have accounted for disease stage by stratifying comparisons into acute, chronic, and AIDS stages for each subtype. We observed that different HIV-1 subtypes accumulate distinct polymorphisms as the disease progresses, as summarized in Table 2 and in the discussion section. Please see Table 2 and lines 437-442.

**Comment #4:** A 200 ns molecular dynamics run may not adequately capture all relevant long-timescale conformational changes.

**Reply**: We thank the reviewer for his valuable comment. While 200 ns molecular dynamics simulations are commonly sufficient to capture the equilibration and stability of protein-RNA complexes, the authors agree that longer simulations can further validate the robustness of the observed trends. Therefore, the authors performed extended molecular dynamics simulations of 500 ns for two representative systems: the A6-TAR complex (strongest binding) and the A1-TAR complex (weakest binding). The extended trajectories exhibited stable RMSD profiles and consistent MM-GBSA binding energies (Δ*G*_binding_ = −140.6 kcal/mol for A6-TAR and −73.4 kcal/mol for A1-TAR), in agreement with the trends observed in the 200 ns MDS. These results confirmed that the conclusions derived from the 200 ns trajectories remain valid. Please see figure S2, and lines 218-223, 290-295, and 314-318.

**Comment #5**: Generating consensus sequences might overlook intra-subtype diversity and rare but functionally significant variants.

**Reply**: We acknowledge that the consensus-sequence approach can mask specific mutations within a single subtype. To address this concern, we have built out a consensus using a threshold of more than 50% amino acid frequency at each position. We found that a substantial sequence conservation across all 9 subtypes: 90% of amino acid residues met the 50% threshold, ranging from 60-95%. The remaining amino acid residues (10%) exhibited variability, but none of these polymorphic sites corresponded to previously investigated polymorphisms that influence Tat-TAR binding affinity. Please see lines 158-165.

**Comment #6**: While the authors acknowledge their limitations in the discussion, it should be emphasized that all findings are based on in silico analyses, which can restrict direct biological or clinical relevance. They should clearly indicate that these results are predictions that require further validation.

**Reply**: We thank the reviewer for this important clarification. We agree that our study needs experimental validation. Accordingly, we have expanded the limitations section to address this point. Please see lines 453-458.
